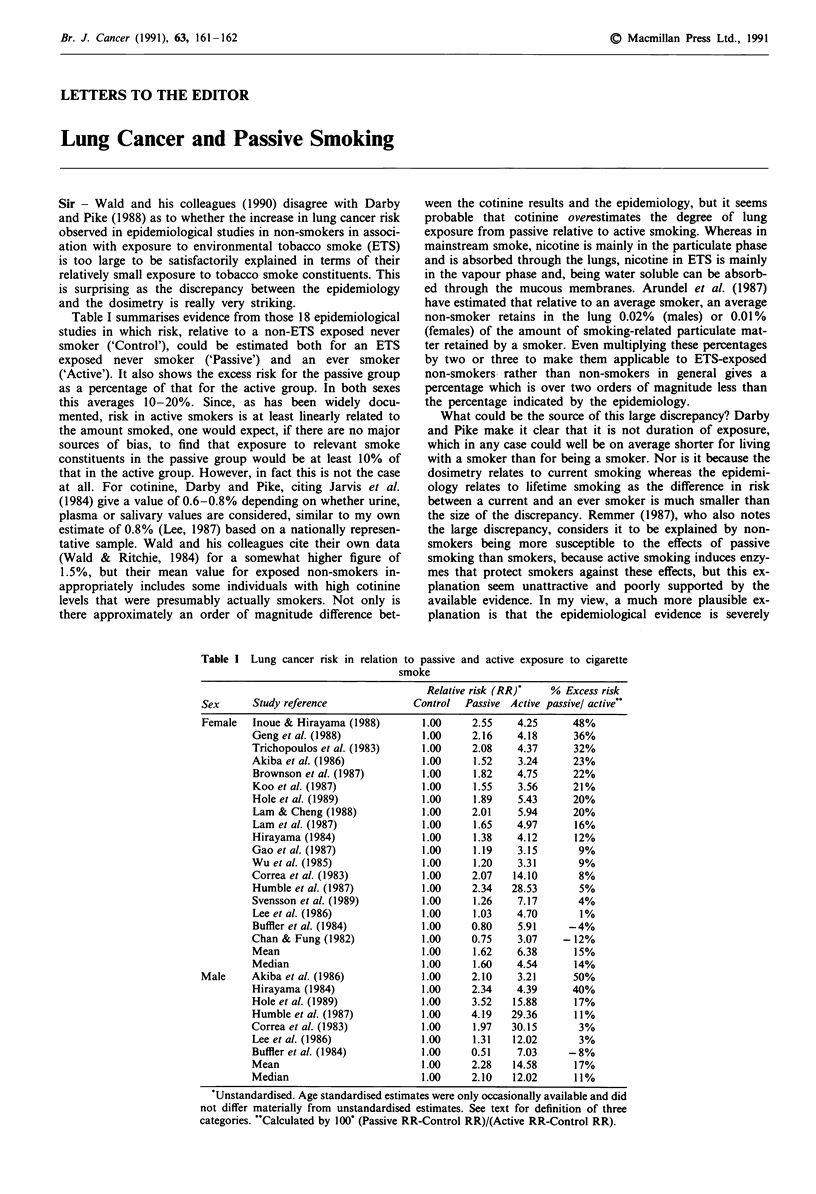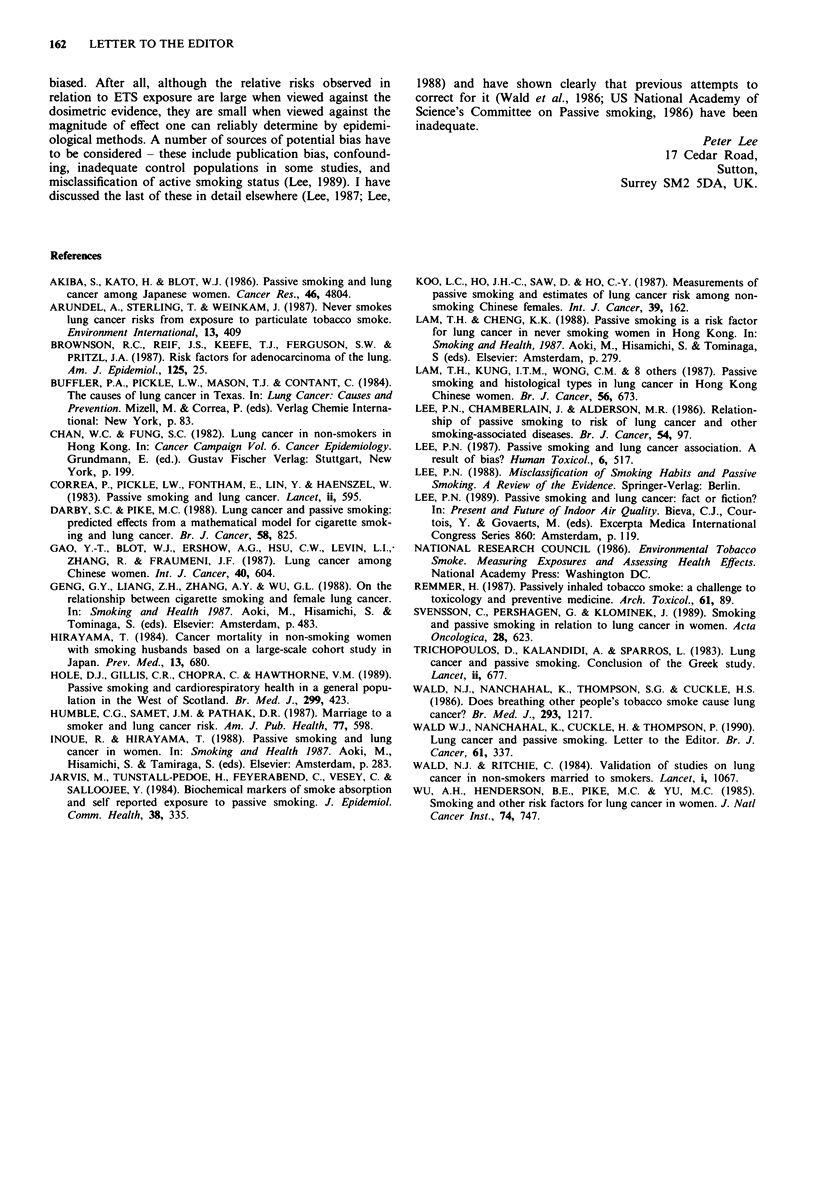# Lung cancer and passive smoking.

**DOI:** 10.1038/bjc.1991.37

**Published:** 1991-01

**Authors:** P. Lee


Br. J. Cancer (1991), 63, 161–162 © Macmillan Press Ltd., 1991

### LETTERS TO THE EDITOR

### Lung Cancer and Passive Smoking

**Sir** – Wald and his colleagues (1990) disagree with Darby and Pike (1988) as to whether the increase in lung cancer risk observed in epidemiological studies in non-smokers in association with exposure to environmental tobacco smoke (ETS) is too large to be satisfactorily explained in terms of their relatively small exposure to tobacco smoke constituents. This is surprising as the discrepancy between the epidemiology and the dosimetry is really very striking.

Table I summarises evidence from those 18 epidemiological studies in which risk, relative to a non-ETS exposed never smoker ('Control'), could be estimated both for an ETS exposed never smoker ('Passive') and an ever smoker ('Active'). It also shows the excess risk for the passive group as a percentage of that for the active group. In both sexes this averages 10–20%. Since, as has been widely documented, risk in active smokers is at least linearly related to the amount smoked, one would expect, if there are no major sources of bias, to find that exposure to relevant smoke constituents in the passive group would be at least 10% of that in the active group. However, in fact this is not the case at all. For cotinine, Darby and Pike, citing Jarvis *et al.* (1984) give a value of 0.6–0.8% depending on whether urine, plasma or salivary values are considered, similar to my own estimate of 0.8% (Lee, 1987) based on a nationally representative sample. Wald and his colleagues cite their own data (Wald & Ritchie, 1984) for a somewhat higher figure of 1.5%, but their mean value for exposed non-smokers inappropriately includes some individuals with high cotinine levels that were presumably actually smokers. Not only is there approximately an order of magnitude difference between the cotinine results and the epidemiology, but it seems probable that cotinine *over*estimates the degree of lung exposure from passive relative to active smoking. Whereas in mainstream smoke, nicotine is mainly in the particulate phase and is absorbed through the lungs, nicotine in ETS is mainly in the vapour phase and, being water soluble can be absorbed through the mucous membranes. Arundel *et al.* (1987) have estimated that relative to an average smoker, an average non-smoker retains in the lung 0.02% (males) or 0.01% (females) of the amount of smoking-related particulate matter retained by a smoker. Even multiplying these percentages by two or three to make them applicable to ETS-exposed non-smokers rather than non-smokers in general gives a percentage which is over two orders of magnitude less than the percentage indicated by the epidemiology.

What could be the source of this large discrepancy? Darby and Pike make it clear that it is not duration of exposure, which in any case could well be on average shorter for living with a smoker than for being a smoker. Nor is it because the dosimetry relates to current smoking whereas the epidemiology relates to lifetime smoking as the difference in risk between a current and an ever smoker is much smaller than the size of the discrepancy. Remmer (1987), who also notes the large discrepancy, considers it to be explained by non-smokers being more susceptible to the effects of passive smoking than smokers, because active smoking induces enzymes that protect smokers against these effects, but this explanation seem unattractive and poorly supported by the available evidence. In my view, a much more plausible explanation is that the epidemiological evidence is severely

**Table I** Lung cancer risk in relation to passive and active exposure to cigarette smoke

| *Sex* | *Study reference* | *Relative risk (RR)** *Control* | *Passive* | *Active* | *% Excess risk passive/ active*** |
|---|---|---|---|---|---|
| Female | Inoue & Hirayama (1988) | 1.00 | 2.55 | 4.25 | 48% |
| | Geng *et al.* (1988) | 1.00 | 2.16 | 4.18 | 36% |
| | Trichopoulos *et al.* (1983) | 1.00 | 2.08 | 4.37 | 32% |
| | Akiba *et al.* (1986) | 1.00 | 1.52 | 3.24 | 23% |
| | Brownson *et al.* (1987) | 1.00 | 1.82 | 4.75 | 22% |
| | Koo *et al.* (1987) | 1.00 | 1.55 | 3.56 | 21% |
| | Hole *et al.* (1989) | 1.00 | 1.89 | 5.43 | 20% |
| | Lam & Cheng (1988) | 1.00 | 2.01 | 5.94 | 20% |
| | Lam *et al.* (1987) | 1.00 | 1.65 | 4.97 | 16% |
| | Hirayama (1984) | 1.00 | 1.38 | 4.12 | 12% |
| | Gao *et al.* (1987) | 1.00 | 1.19 | 3.15 | 9% |
| | Wu *et al.* (1985) | 1.00 | 1.20 | 3.31 | 9% |
| | Correa *et al.* (1983) | 1.00 | 2.07 | 14.10 | 8% |
| | Humble *et al.* (1987) | 1.00 | 2.34 | 28.53 | 5% |
| | Svensson *et al.* (1989) | 1.00 | 1.26 | 7.17 | 4% |
| | Lee *et al.* (1986) | 1.00 | 1.03 | 4.70 | 1% |
| | Buffler *et al.* (1984) | 1.00 | 0.80 | 5.91 | –4% |
| | Chan & Fung (1982) | 1.00 | 0.75 | 3.07 | –12% |
| | Mean | 1.00 | 1.62 | 6.38 | 15% |
| | Median | 1.00 | 1.60 | 4.54 | 14% |
| Male | Akiba *et al.* (1986) | 1.00 | 2.10 | 3.21 | 50% |
| | Hirayama (1984) | 1.00 | 2.34 | 4.39 | 40% |
| | Hole *et al.* (1989) | 1.00 | 3.52 | 15.88 | 17% |
| | Humble *et al.* (1987) | 1.00 | 4.19 | 29.36 | 11% |
| | Correa *et al.* (1983) | 1.00 | 1.97 | 30.15 | 3% |
| | Lee *et al.* (1986) | 1.00 | 1.31 | 12.02 | 3% |
| | Buffler *et al.* (1984) | 1.00 | 0.51 | 7.03 | –8% |
| | Mean | 1.00 | 2.28 | 14.58 | 17% |
| | Median | 1.00 | 2.10 | 12.02 | 11% |

*Unstandardised. Age standardised estimates were only occasionally available and did not differ materially from unstandardised estimates. See text for definition of three categories. **Calculated by 100* (Passive RR-Control RR)/(Active RR-Control RR).

biased. After all, although the relative risks observed in relation to ETS exposure are large when viewed against the dosimetric evidence, they are small when viewed against the magnitude of effect one can reliably determine by epidemiological methods. A number of sources of potential bias have to be considered – these include publication bias, confounding, inadequate control populations in some studies, and misclassification of active smoking status (Lee, 1989). I have discussed the last of these in detail elsewhere (Lee, 1987; Lee, 1988) and have shown clearly that previous attempts to correct for it (Wald *et al.*, 1986; US National Academy of Science's Committee on Passive smoking, 1986) have been inadequate.

*Peter Lee*
17 Cedar Road,
Sutton,
Surrey SM2 5DA, UK.

### References

AKIBA, S., KATO, H. & BLOT, W.J. (1986). Passive smoking and lung cancer among Japanese women. *Cancer Res.*, **46**, 4804.

ARUNDEL, A., STERLING, T. & WEINKAM, J. (1987). Never smokes lung cancer risks from exposure to particulate tobacco smoke. *Environment International*, **13**, 409

BROWNSON, R.C., REIF, J.S., KEEFE, T.J., FERGUSON, S.W. & PRITZL, J.A. (1987). Risk factors for adenocarcinoma of the lung. *Am. J. Epidemiol.*, **125**, 25.

BUFFLER, P.A., PICKLE, L.W., MASON, T.J. & CONTANT, C. (1984). The causes of lung cancer in Texas. In: *Lung Cancer: Causes and Prevention*. Mizell, M. & Correa, P. (eds). Verlag Chemie International: New York, p. 83.

CHAN, W.C. & FUNG, S.C. (1982). Lung cancer in non-smokers in Hong Kong. In: *Cancer Campaign Vol. 6. Cancer Epidemiology*. Grundmann, E. (ed.). Gustav Fischer Verlag: Stuttgart, New York, p. 199.

CORREA, P., PICKLE, LW., FONTHAM, E., LIN, Y. & HAENSZEL, W. (1983). Passive smoking and lung cancer. *Lancet*, **ii**, 595.

DARBY, S.C. & PIKE, M.C. (1988). Lung cancer and passive smoking: predicted effects from a mathematical model for cigarette smoking and lung cancer. *Br. J. Cancer*, **58**, 825.

GAO, Y.-T., BLOT, W.J., ERSHOW, A.G., HSU, C.W., LEVIN, L.I., ZHANG, R. & FRAUMENI, J.F. (1987). Lung cancer among Chinese women. *Int. J. Cancer*, **40**, 604.

GENG, G.Y., LIANG, Z.H., ZHANG, A.Y. & WU, G.L. (1988). On the relationship between cigarette smoking and female lung cancer. In: *Smoking and Health 1987*. Aoki, M., Hisamichi, S. & Tominaga, S. (eds). Elsevier: Amsterdam, p. 483.

HIRAYAMA, T. (1984). Cancer mortality in non-smoking women with smoking husbands based on a large-scale cohort study in Japan. *Prev. Med.*, **13**, 680.

HOLE, D.J., GILLIS, C.R., CHOPRA, C. & HAWTHORNE, V.M. (1989). Passive smoking and cardiorespiratory health in a general population in the West of Scotland. *Br. Med. J.*, **299**, 423.

HUMBLE, C.G., SAMET, J.M. & PATHAK, D.R. (1987). Marriage to a smoker and lung cancer risk. *Am. J. Pub. Health*, **77**, 598.

INOUE, R. & HIRAYAMA, T. (1988). Passive smoking and lung cancer in women. In: *Smoking and Health 1987*. Aoki, M., Hisamichi, S. & Tamiraga, S. (eds). Elsevier: Amsterdam, p. 283.

JARVIS, M., TUNSTALL-PEDOE, H., FEYERABEND, C., VESEY, C. & SALLOOJEE, Y. (1984). Biochemical markers of smoke absorption and self reported exposure to passive smoking. *J. Epidemiol. Comm. Health*, **38**, 335.

KOO, L.C., HO, J.H.-C., SAW, D. & HO, C.-Y. (1987). Measurements of passive smoking and estimates of lung cancer risk among non-smoking Chinese females. *Int. J. Cancer*, **39**, 162.

LAM, T.H. & CHENG, K.K. (1988). Passive smoking is a risk factor for lung cancer in never smoking women in Hong Kong. In: *Smoking and Health, 1987*. Aoki, M., Hisamichi, S. & Tominaga, S (eds). Elsevier: Amsterdam, p. 279.

LAM, T.H., KUNG, I.T.M., WONG, C.M. & 8 others (1987). Passive smoking and histological types in lung cancer in Hong Kong Chinese women. *Br. J. Cancer*, **56**, 673.

LEE, P.N., CHAMBERLAIN, J. & ALDERSON, M.R. (1986). Relationship of passive smoking to risk of lung cancer and other smoking-associated diseases. *Br. J. Cancer*, **54**, 97.

LEE, P.N. (1987). Passive smoking and lung cancer association. A result of bias? *Human Toxicol.*, **6**, 517.

LEE, P.N. (1988). *Misclassification of Smoking Habits and Passive Smoking. A Review of the Evidence*. Springer-Verlag: Berlin.

LEE, P.N. (1989). Passive smoking and lung cancer: fact or fiction? In: *Present and Future of Indoor Air Quality*. Bieva, C.J., Courtois, Y. & Govaerts, M. (eds). Excerpta Medica International Congress Series 860: Amsterdam, p. 119.

NATIONAL RESEARCH COUNCIL (1986). *Environmental Tobacco Smoke. Measuring Exposures and Assessing Health Effects*. National Academy Press: Washington DC.

REMMER, H. (1987). Passively inhaled tobacco smoke: a challenge to toxicology and preventive medicine. *Arch. Toxicol.*, **61**, 89.

SVENSSON, C., PERSHAGEN, G. & KLOMINEK, J. (1989). Smoking and passive smoking in relation to lung cancer in women. *Acta Oncologica*, **28**, 623.

TRICHOPOULOS, D., KALANDIDI, A. & SPARROS, L. (1983). Lung cancer and passive smoking. Conclusion of the Greek study. *Lancet*, **ii**, 677.

WALD, N.J., NANCHAHAL, K., THOMPSON, S.G. & CUCKLE, H.S. (1986). Does breathing other people's tobacco smoke cause lung cancer? *Br. Med. J.*, **293**, 1217.

WALD W.J., NANCHAHAL, K., CUCKLE, H. & THOMPSON, P. (1990). Lung cancer and passive smoking. Letter to the Editor. *Br. J. Cancer*, **61**, 337.

WALD, N.J. & RITCHIE, C. (1984). Validation of studies on lung cancer in non-smokers married to smokers. *Lancet*, **i**, 1067.

WU, A.H., HENDERSON, B.E., PIKE, M.C. & YU, M.C. (1985). Smoking and other risk factors for lung cancer in women. *J. Natl Cancer Inst.*, **74**, 747.